# Compliant Glass Mechanism Instrumented with a Bragg Grating to Measure Indentation Force

**DOI:** 10.3390/mi17050572

**Published:** 2026-05-05

**Authors:** Manon Marchandise, Adam Chafai, Christophe Caucheteur, Pierre Lambert

**Affiliations:** 1 TIPs Department (CP 165/67), Brussels School of Engineering, Solbosch Campus, Université Libre de Bruxelles, Avenue F.D. Roosevelt 50, B-1050 Brussels, Belgiumadam.chafai@ulb.be (A.C.); 2 Service d’Electromagnétisme et Télécommunications, Polytech Mons, Bâtiment Dolez, Université de Mons, Boulevard Dolez 31, B-7000 Mons, Belgium; christophe.caucheteur@umons.ac.be

**Keywords:** lung tissues, stiffness, Bragg grating, compliant mechanism, force sensor, micro-indentation

## Abstract

This paper presents a force sensor made of a compliant glass mechanism instrumented with a waveguide and a Bragg grating, measuring the reflected wavelength shift produced by the strain in the compliant element generated by the applied force. The compliant element geometry and material have been chosen for the sensor to be spliced or manufactured at the extremity of an optical fiber, enabling possible insertion of the instrument in the bronchial tree after embedding in a proper catheter. The context of this research is the mechanical discrimination between healthy and cancerous lung tissues based on their mechanical signature. The paper proposes a comprehensive study including the mechanical design of the structure and the optimization of the production parameters, thanks to an experimental parametric study. After experimental characterization of the mechanism stiffness, the optical response to a mechanical force is reproduced with two different samples on two different days (more than 25 repetitions). The conclusion is that a fair linear and repeatable response is observed (±26 mN) for forces ranging from 0 to 250 mN.

## 1. Introduction

This paper presents a new force sensor in the form of an indentation tip that paves the way for mechanical discrimination of healthy vs. cancerous lung tissues by indentation.

Lung cancer represented 2.5 million new cases worldwide in 2022 [1]. It is the deadliest cancer, representing 18% of all cancer deaths. Early diagnosis is crucial to increase the survival rate at 5 years, from 65% for patients diagnosed at stage I to only 5% at stage IV. Nowadays, a biopsy is required to confirm the diagnosis, while most nodules are difficult to access, as they are located in the so-called lung periphery [2]. Therefore, thinner sensors or those mounted on bronchoscopes could help reduce the number of biopsies or even provide an alternative when the targeted tissues cannot be reached by biopsy tools. In vivo sensors are excellent alternatives, but biochemical sensors cannot give accurate spatial information, since the binding of proteins and antibodies happens all along the anatomical pathway inside the patient. Contrarily, mechanical indentation sensors could circumvent this difficulty by offering a localized, fast indentation force measurement. Beyond lungs, such a tool would help characterize many other tissues, such as the bladder, endometrial tissue in gynecology, the heart, and digestive tissues, or lymph nodes. Even for organs such as the prostate or tongue, which are relatively easier to access, a mechanical diagnostic could help to better target the biopsies. Ref. [3] indeed reports the modulus ratio between normal and cancerous cells using an atomic force microscope for various tissues. More generally, mechanical properties are known to be a key marker for cell growth [4].

The goal of this research, as exposed in this paper, is to design and develop a new sensor for insertion in the operating channel of a bronchoscope (inner diameter down to 1.2 mm) to mechanically characterize lung tissue in vivo. This paper reports only preliminary results on the force-sensing element, equipped with a waveguide and a Bragg grating in order to measure the force applied to the fiber tip during its insertion into tissues.

The assumption is that this force can be converted into tissue stiffness, further used to discriminate between healthy and tumoral tissues.

**Figure 1:**
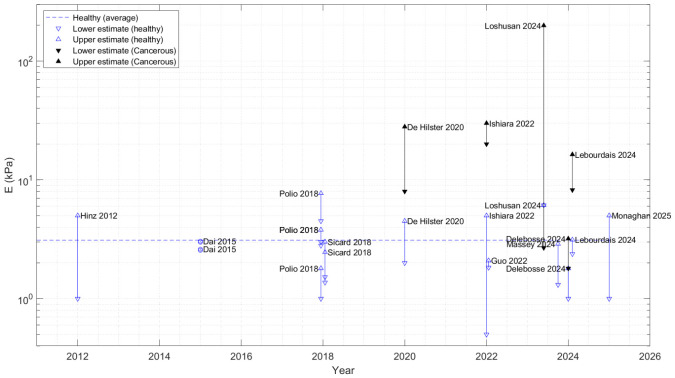
Young modulus for healthy and cancerous lung tissues. Average *E* modulus for healthy tissues is about 3 kPa, while most cancerous tissues are stiffer (up to 2 orders of magnitude). ’o’ is the symbol of tests made on a pig. Sources: [6,7,10,11,12,13,14,15,16,17,18,19,20].

Scientifically, the mechanical characterization of lung tissues is important for optimizing ventilation treatment [5], monitoring of arterial hypertension and pulmonary fibrosis [6], or optimizing the culture media with adequate stiffness to develop new drugs against lung cancer mutations [7]. Adapted from [8], Figure 1 displays healthy tissues’ stiffness (Young modulus) ranging between 1 and 5 kPa, while cancerous tissues are up to five times stiffer according to [9].

Several comments arise from these data. First, while cancerous tissues are stiffer than healthy tissues, cancer cells are softer than healthy ones, contributing to measurement dispersion. Secondly and consequently, the scale and therefore the measurement technique impact the results. At the macro-scale, mechanical properties of the lungs are measured with volume-pressure measurements, punch indentation, or tensile testing of isolated biopsied tissues [15]. At smaller scales, punch indentation is replaced by AFM indentation with micrometer-size tips [6]. In addition to indentation, magnetic resonance elastography is promising, but it is not yet an established method in bronchoscopy [6].

Thirdly, lung tissue stiffness also depends on the respiration cycle through the transpulmonary pressure [9]. Finally, the post-processing of the indentation data also impacts the results. In the literature, Young modulus *E* is typically deduced from contact force measurement (Hertz contact mechanics).

**Figure 2:**
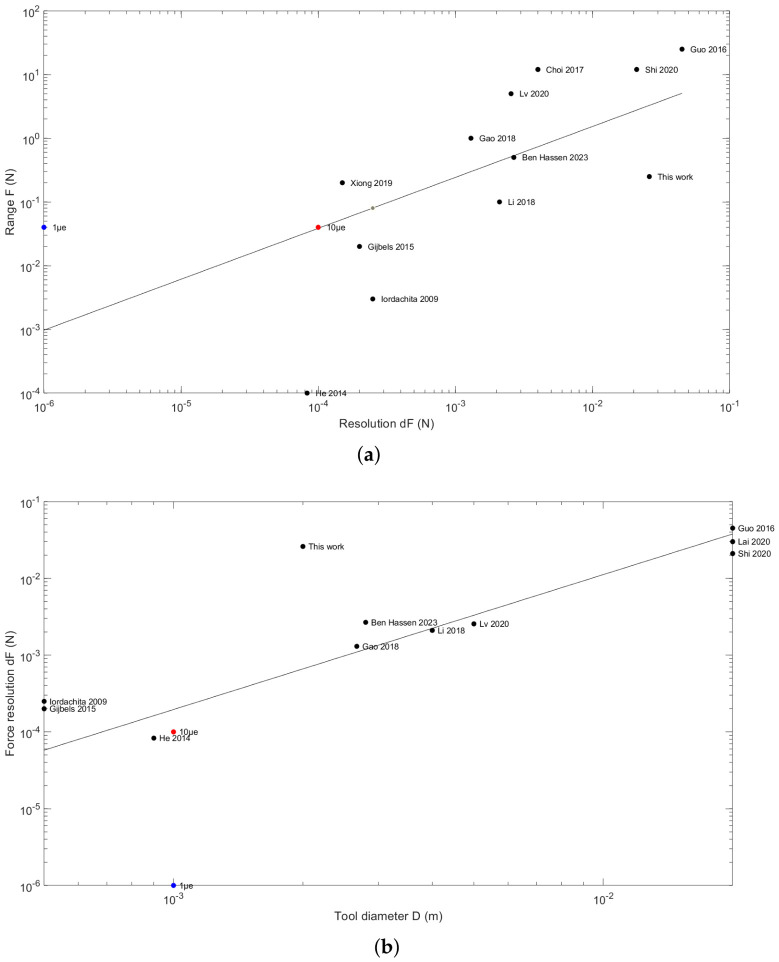
The literature comparison of Bragg grating instrumented surgical tools (adapted from [21]) and the perspectives with a standard wavelength shift analyzer (1pm, red marker) or 10 times more resolute (blue marker). (**a**) Maximal force range *F* as a function of the force measurement resolution dF, scaling law $F=4dF^{1.6}$. (**b**) Comparison of force sensors in medical applications: force resolution dF as a function of the tool diameter *D*, scaling law $dF=0.9D^{1.4}$. Sources: [21,22,23,24,25,26,27,28,29,30,31].

This paper relies on the use of Bragg gratings for embedded force sensing in medical instruments. Ref. [21] summarizes the benefits of such systems in medical applications (sensitivity, biocompatibility, chemical inertness, and insensitivity to magnetic field) and reports many application fields (endovascular intervention in the cardiac area, retinal microsurgery, prostate intervention, laparoscopic and robotic surgery, and flexible endoscopy). Most architectures combine three to five fibers (typically one Bragg grating for temperature auto-calibration and three of them for the three degrees-of-freedom: one compression and two bendings), disposed around a central or external compliant structure made of aluminum flexure, hollow stainless-steel tube, titanium wire shaft, or hollow nitinol tube with cuts. These designs are limited by the materials of the compliant structure and by the high level of assembly complexity. The material limitation is given by the maximal elastic strain σ/E ratio: based on this criterion, glass is better than titanium alloys, steel, aluminum, or nitinol. Figure 2 summarizes the main features of these Bragg grating-based solutions found in the literature.

The second element is the current trend to use monolithic mechanisms, i.e., produced in a single-step production without assembly, as reported by, for instance, ref. [32] for a vitrectomy mechanism in eye surgery.

Finally, glass shaping at a microscale is considerably improved thanks to femtosecond laser pulse processing, allowing the fine production of 3D structures in glass parts. The illumination of glass first leads to an increase in the refractive index, and at a higher exposure dose, to a local densification surrounded by a zone with tensile stress, promoting a higher etching rate in KOH [33]. At a larger tensile stress, the appearance of cracks limits the etching rate. This allows the precise shaping of glass. In the medical domain, it has enabled the emergence of bistable mechanisms limiting the stroke, such as puncturing needles in vein canulation [34].

More specifically, in terms of femtosecond laser pulses engineering of glass, it has been demonstrated that three types of refractive index modulations can be generated depending on the femtosecond laser pulses properties [35,36,37,38,39], i.e., pulse energy and duration, repetition rate, scanning speed and direction, and polarization. With increasing energy density levels, the silica glass undergoes different structural changes: (1) continuous densification enabling an increase in the refractive index to produce optical waveguides; (2) micro/nano gratings or shaping (ablation) upon subsequent exposure to an etchant chemical; and (3) formation of voids. All these changes have already been successfully implemented, as reported in [40,41].

**Figure 3:**
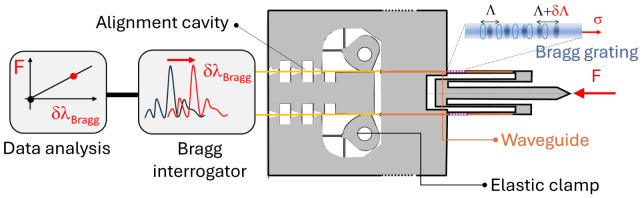
A force *F* is applied onto the tip of a compliant mechanism. The induced deformation is measured thanks to the wavelength shift in a light beam reflected by a Bragg grating patterned in the strained area. The reflected spectrum is monitored by a Bragg interrogator, and the wavelength shift δλ is further processed into the value of the applied force, after stiffness calibration of the mechanism.

The main idea followed in this paper is shown in Figure 3. Under the application of an external force, the compliant structure, further described in Section 2.1 deforms according to the model presented in Section 2.2. Thanks to the fabrication and characterization processes explained in Section 2.3 and Section 2.4, the following results could be obtained (Section 3): mechanical stiffness of the mechanism and its optical response to a mechanical force, thanks to the analysis of wavelength shift in a light beam reflected by a Bragg grating engraved in the compliant part of the tip. The observed limitations are discussed in Section 4 and conclusions are drawn in Section 5.

## 2. Materials and Methods

### 2.1. Mechanical Principle

**Figure 4:**
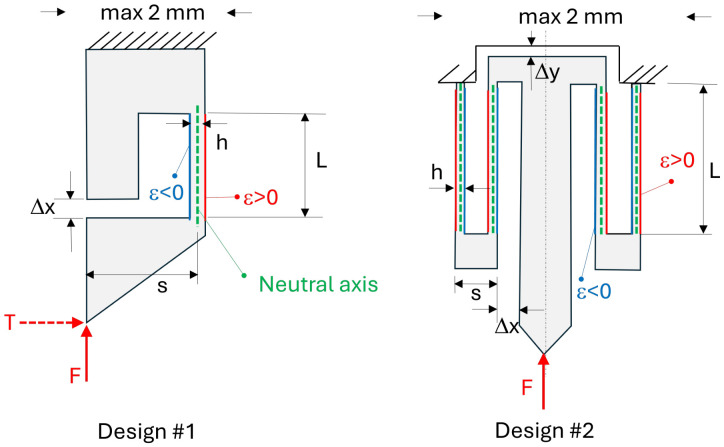
Design 1 is non-symmetric: upon application of *F*, the tip exhibits a shear displacement and undergoes a shear force *T*. Design 2 deforms symmetrically upon application of *F*. Parameters *h* and *L* are respectively the flexible beam thickness and length. Δx represents the displacement until a mechanical stop (preventing excessive displacements). *s* acts as the lever arm of the applied force *F*. The max lateral size is chosen at 2mm to be compatible with most endoscopic channels.

Figure 4 provides two successive designs. Design #1 converts the force *F* applied upwards to the tip into a bending deformation of the vertical thin beam of thickness *h* and length *L*. The force lever arm has a length *s*. The out-of-plane dimension *b* (not represented) depends on the glass slide thickness used to produce the samples (see later on). The right side of this beam undergoes traction (ϵ>0 in red) while the left side undergoes compression (ϵ<0 in blue). Consequently, since the sample is in glass, it can be instrumented with a waveguide and a Bragg grating patterned in the non-zero ϵ area. Plugging an optical fiber in this instrument (aligned with the waveguide) enables its connection to a Bragg spectrum meter for reading the strain level. Thanks to the mechanical model or calibration, this strain can further be converted into the force *F* applied to the tip. As further explained, this design #1 is subjected to a transverse force *T* when the tip is applied to or inserted in a substrate.

A second design (design #2) has thus been designed symmetrically. The geometry of these designs is explained in Figure 4 and Table 1.

### 2.2. Mechanical Design

**Figure A1:**
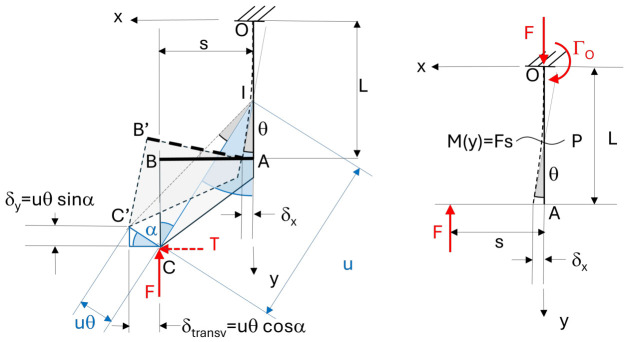
Mechanical model (design #1).

**Figure A3:**
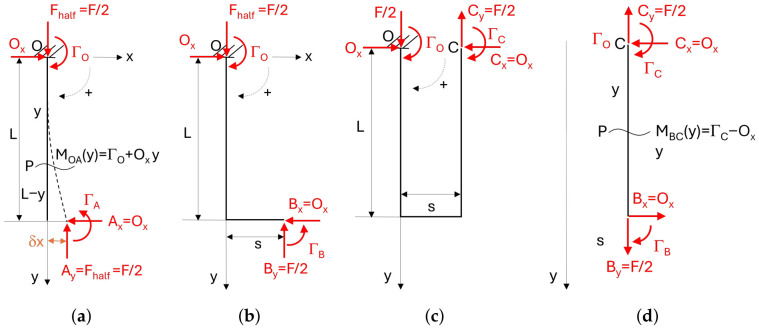
Forces diagrams of (**a**) OA beam; (**b**) OB beam; (**c**) OC beam; (**d**) BC beam.

The mechanical design is based on the well-known Euler–Bernoulli theory, limited to the linear regime. The general sketch and a more precise forces representation are given in Figure A1 for design #1 and Figure A3 for design #2.

In both cases, the stiffness is defined as the ratio between the force *F* applied to the sharp tip and the resulting displacement $\delta_{y}$ (details are provided in Appendix A and Appendix B)

(1) $$k_{1}=\frac{F}{\delta_{y}}\;=\;\frac{EI}{s^{2}L}$$

(2) $$k_{2}=\frac{F}{\delta_{y}}\;=\;\frac{4EI}{s^{2}L}$$

### 2.3. Fabrication

#### 2.3.1. Mechanical Fabrication

The raw material is made of fused silica glass slides (Siegert Wafer GmbH, Aachen, Germany $74\times 26\;mm^{2}\;\pm \;0.1\;mm$ with a thickness b=500 µm ± 20, Ra < 1 nm). Preliminary characterization reported in [40] indicates, in similar conditions, a Young modulus equal to E=72GPa and an ultimate stress about σ=1GPa, therefore exhibiting a remarkable elastic coefficient σ/E and making glass a unique candidate for compliant mechanisms. Due to its biocompatibility, fused silica can also be used in the medical field.

To shape the compliant mechanism out of the silica glass slide, a femtosecond laser-assisted (wet) etching of fused silica has been used, combining a first femtosecond laser irradiation followed by an etching step in a 12 M KOH solution at 85 °C [40]. The process parameters are those reported in [41] with the × 20 objective: an energy pulse of 230 nJ, a repetition rate equal to 1000 kHz, and a writing speed equal to 950 mm/min (with perpendicular polarization). For these values, ref. [41] reports a differential etching rate equal to 130 µm/h for the illuminated area against 0.7 µm/h for the non-illuminated regions. These values are summarized in Table 2.

**Figure 5:**
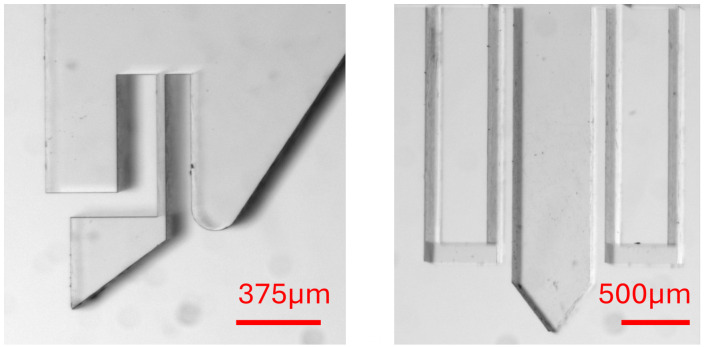
Geometrical result of the fabrication step (the scale corresponds to the geometrical parameters given in Table 1).

Examples of microscope images for designs #1 and #2 are shown in Figure 5.

#### 2.3.2. Waveguides

The production of waveguides in fused silica with a femtosecond laser has been described in [41]. The exact values of the machine parameters (energy pulse, writing speed, and pitch) have been optimized (with the method of design of experiments) as given in Appendix C. As a result, the combination of parameters that maximizes the difference in refractive index is an energy of 150 nJ, a writing speed of 50 mm/min, and a pitch of 0.5µm.

#### 2.3.3. Bragg Gratings

The process parameters towards Bragg gratings inscription are also based on [41]. The energy pulse has been adapted to the same 150 nJ as for the waveguides. The Bragg period was chosen Λ=1.1µm, corresponding (for an order m=2) to a reflection spectrum centered on

(3) $$\lambda_{\mathrm{Bragg}}=\frac{2n_{\mathrm{eff}}\Lambda }{m}=1589.5\;nm$$

and consequently a relationship between the shift $\Delta \lambda_{\mathrm{Bragg}}$ and the strain ϵ given by

(4) $$\Delta \lambda_{\mathrm{Bragg}}=\lambda_{\mathrm{Bragg}}\epsilon \left(1-p_{e}\right)=1.24\epsilon$$

where $p_{e}=0.22$ is the photo-elastic coefficient of fused silica and the value 1.24 obtained for ϵ expressed in pm/µϵ.

### 2.4. Characterization

#### 2.4.1. Mechanical Analysis Towards the Stiffness

**Figure 6:**
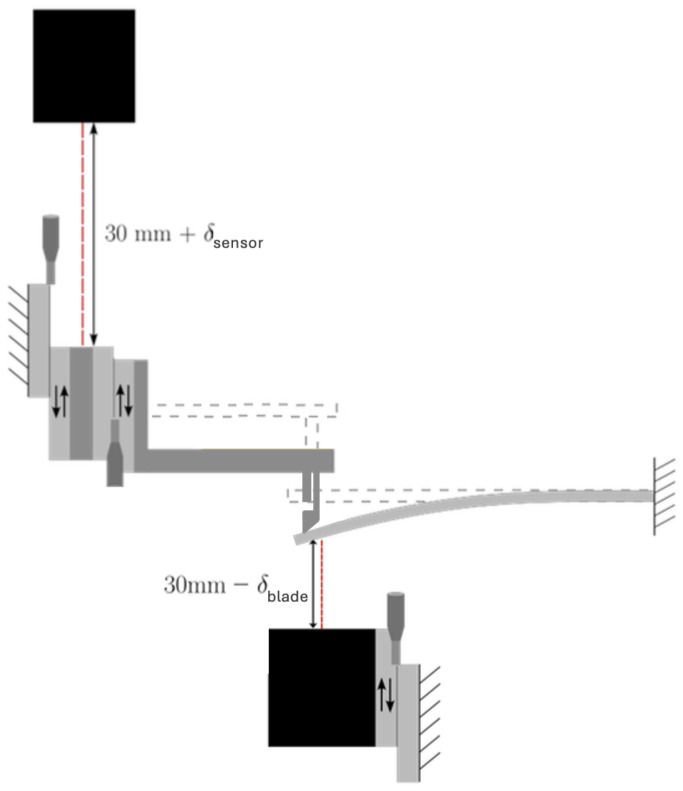
Mechanical setup for the characterization of stiffness (illustrated for design #1).

**Figure 7:**
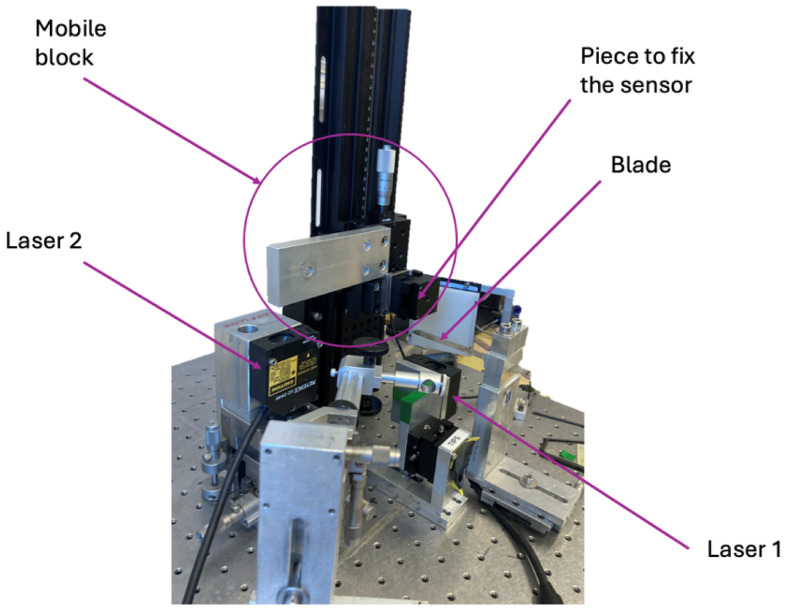
Mechanical setup for the characterization of stiffness (corresponding picture).

The stiffness was measured on a home-made setup shown in Figure 6 and Figure 7. The glass structure (on the left) is mounted on a manual stage whose position is tracked with a non-contact displacement sensor (Keyence LC-2440, Osaka, Japan). An external force is applied to the mechanism tip by contacting it with a high-carbon spring steel cantilevered beam (Precision Brand 09740-20 Piece Metric Steel Feeler Gage Poc-kit Asst 12.7 mm × 127 mm Blades, Downers Grove, IL, USA) with calibrated stiffness $k_{\mathrm{cal}}$. Its displacement (hence the applied force) is measured with a second non-contact displacement laser. The applied force is given by

(5) $$F=k_{\mathrm{cal}}\delta_{\mathrm{blade}}$$

and the corresponding displacement $\delta_{y}$ of the glass tip is given by

(6) $$\delta_{y}=\delta_{\mathrm{sensor}}-\delta_{\mathrm{blade}}$$

Consequently, the stiffness of the compliant designs is given by

(7) $$k_{1,2}=\frac{k_{\mathrm{cal}}\delta_{\mathrm{blade}}}{\delta_{\mathrm{sensor}}-\delta_{\mathrm{blade}}}$$

#### 2.4.2. Optical Analysis Towards the Bragg Shift

**Figure 8:**
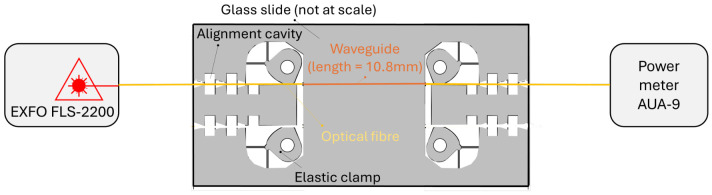
Sketch of the setup used to assess the coupling and transmission losses.

First, the coupling and propagation losses were estimated as follows: a dedicated glass slide was prepared including an input and output made of alignment cavities and elastic clamps, connected by a 10.8 mm long waveguide which is about 2 times larger than the travel length between the beginning of the waveguide and the position of the Bragg grating in the final sensor (Figure 8). The input was connected to an optical source EXFO model FLS2200, Eastleigh, UK (1 mW or 0 dBm) and the output to a power meter (AUA-9 Power Meter 850–1625 nm FC-PC). With the selected process parameters (speed = 50 mm/min, energy = 150 nJ, and pitch = 0.5µm), the transmitted power was equal to 0.070 mW (−11.54 dBm). This measure confirms the inscription of the waveguide (in the absence of a waveguide in the glass slide, the transmitted power is 30 times smaller) and the choice of the writing speed at 50 mm/min (by comparison, a waveguide printed with 30 mm/min only transmits 0.061 mW).

Two different samples of design #2 (labeled 4 and 6) were then connected to a Bragg interrogator (brand fibersensing featuring a tunable laser source, a photodetector, and a circulator to acquire the reflected amplitude spectrum from the Bragg grating) with a Thorlabs GF1 optical fiber (1500–1600 nm with a cladding diameter equal to 125µm ± 1.5). A standard single-mode optical fiber pigtail makes the connection between the interrogator and the instrumented compliant structure. Index matching gel G608N3 (Thorlabs, Newton, NJ, USA) has been used at the interface between the optical fiber and the waveguide.

To assess the robustness of the coupling, the linear power peak of the reflected Bragg spectra was compared for zero load, reporting 12.9 and 13.2 µW for the sample 4 (P=13.05µW ± 0.21) and 16.5, 21.4, 21.9, and 21.5 µW for the sample 6 (P=20.33µW ± 0.25).

**Figure 9:**
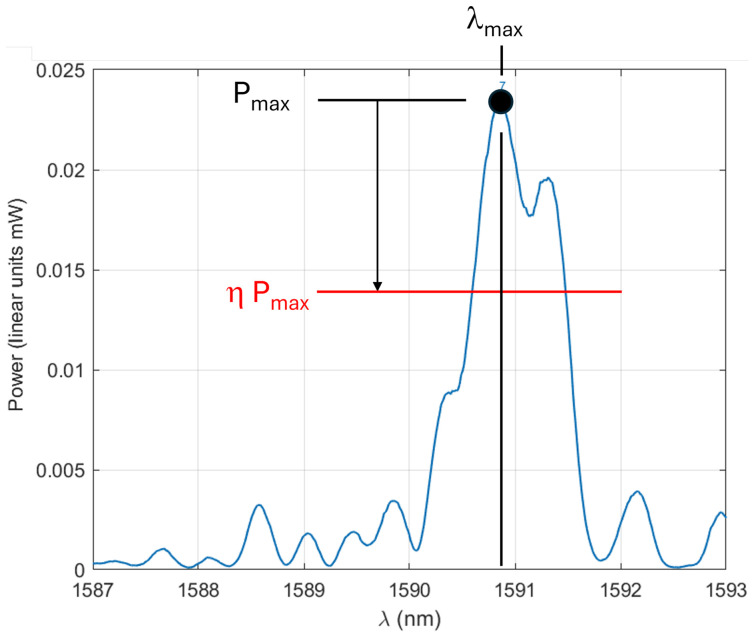
Identification of the position $\lambda_{\max}$ of the power peak $P_{\max}$, further defining the power threshold $\eta P_{\max}$ for the next step of the analysis.

The samples were then submitted to a force ramp ranging from 0 to about 350mN while acquiring the full reflected spectrum for each sample and for each applied force (27 spectra acquired in total). The first step of the analysis is to locate the wavelength $\lambda_{\max}$ corresponding to the power peak (linear units in mW), and to determine a threshold power level for step 2, corresponding to the fraction η of the maximal power (Figure 9). This cut-off enables us to get rid of the noise far away from the peak. The chosen value is η=0.48, minimizing the noise level for the final force resolution (see Appendix D).

**Figure 10:**
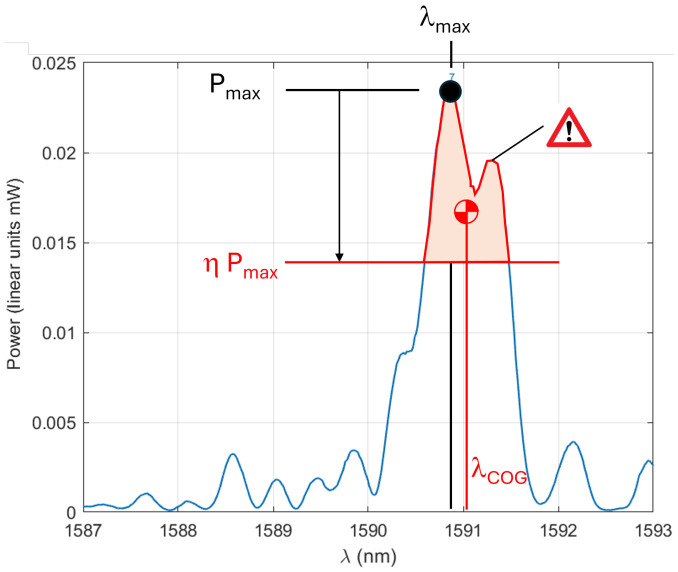
Identification of the position $\lambda_{\mathrm{COG}}$ of the spectrum portion located above the threshold $\eta P_{\max}$.

**Figure 11:**
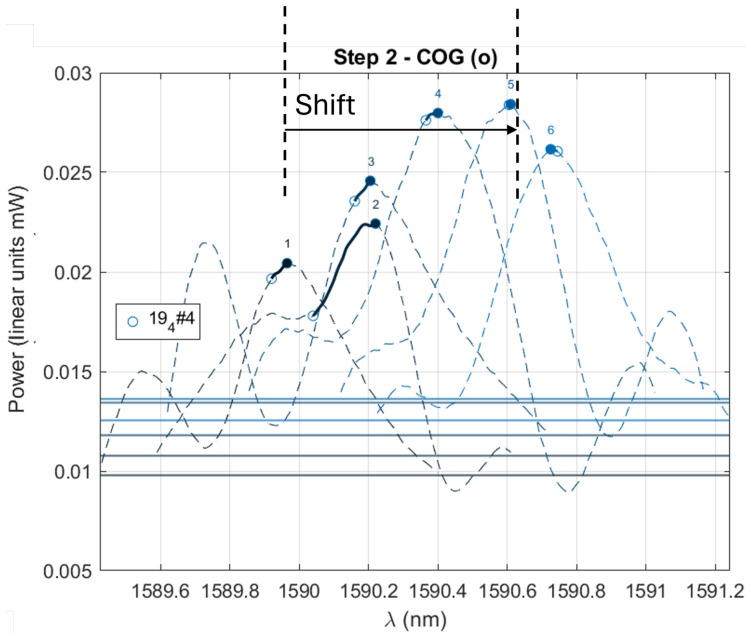
Example of 6 successive spectra (dashed lines numbered from 1 to 6) obtained with increasing load on the glass mechanism. For each spectrum, both the position of the max and the position of $\lambda_{\mathrm{COG}}$ are shown, where $\lambda_{\mathrm{COG}}$ is calculated for the spectrum portion located above the threshold $\eta P_{\max}$. When the spectrum is symmetric, $\lambda_{\mathrm{COG}}=\lambda_{\max}$.

Because a second emerging peak is observed in many cases (likely to be the signature of bi-refringence), the position of the peak does not reflect the Bragg shift correctly. The second step, therefore, consists of calculating the wavelength $\lambda_{\mathrm{COG}}$ of the gravity center of the spectrum portion exhibiting powers above $\eta P_{\max}$ (Figure 10). When the spectrum is perfectly symmetric, $\lambda_{\mathrm{COG}}=\lambda_{\max}$ (Figure 11).

Applying an external force to the mechanical structure varies the strain and, therefore, the reflected wavelength. Hence, the difference between identified peaks’ wavelengths leads to the peak shift (in nm or pm).

## 3. Results

### 3.1. Production of an Instrumented Compliant Mechanism and Its Optical Connection

**Figure 12:**
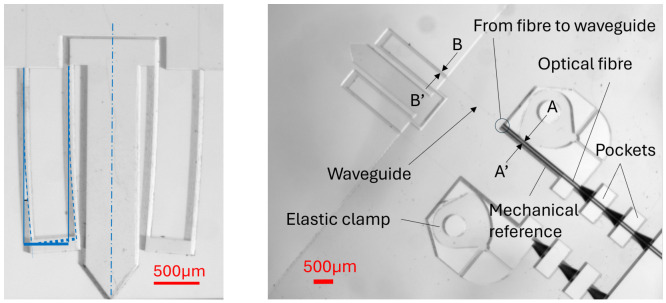
(**Left**) Example of deformed design #2 and (**right**) connection system made of 3 successive pockets connected by an internal channel for the raw position of the optical fiber and elastic clamp (on the right of the figure) for the fine positioning of the optical fiber on the bottom reference surface obtained by manufacturing.

The manufacturing of parts, waveguides, and Bragg gratings was achieved according to the process parameters reported in Table 2. Figure 12 (left) gives an example of symmetric deformation of design #2 under a typical axial load. Figure 12 (right) shows the compliant mechanism and an optical fiber positioned in front of the waveguide, thanks to alignment pockets (for rough positioning) and the combination of a mechanical reference and an elastic clamp for the fine positioning. An index-matching gel is used at the tip of the optical fiber in front of the waveguide (the latter cannot be seen in this figure).

### 3.2. Mechanical Stiffness of Both Designs #1 and #2

**Figure 13:**
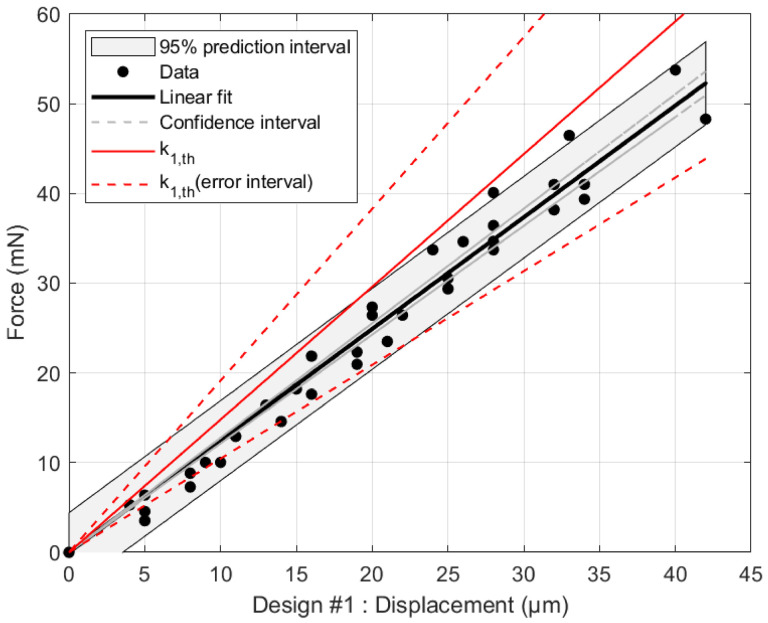
Stiffness of design #1: (in black) measured $k_{1}=1.24\;kNm^{-1}\;\pm \;0.03$ against (in red) calculated $k_{1,\mathrm{th}}=1.48\;kNm^{-1}\;\pm \;0.43$ (considering 3µm error on the lengths and 1% on *E*).

Figure 13 shows the force-displacement characteristics of design #1. Using Equation (1) and the geometrical data of Table 1, the stiffness model provides a stiffness shown by the solid red line, equal to $k_{1,\mathrm{th}}=1.48\;kNm^{-1}\;\pm \;0.43$ (considering 3µm error on the lengths and 1% on *E*). The black points are the measures, whose linear fit leads to an experimental stiffness equal to $1.24\;kNm^{-1}\;\pm \;0.03$. The gray strip represents the prediction interval. Both theoretical and experimental estimations fairly overlap one another.

**Figure 14:**
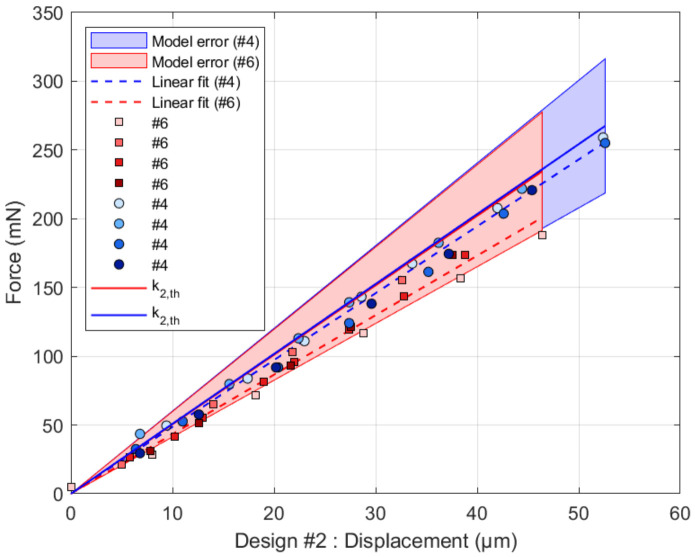
Stiffness of design #2. For sample #4 (circles), the experimental stiffness (dashed blue line) is $k_{2_{\#4}}=4.86\;kNm^{-1}\;\pm \;0.16$ against a calculated $k_{2,{\mathrm{th}}_{\#4}}=5.09\;kNm^{-1}\;\pm \;0.93$. For sample #6 (squares), the experimental stiffness (dashed red line) is $k_{2_{\#4}}=4.33\;kNm^{-1}\;\pm \;0.23$ against a calculated $k_{2,{\mathrm{th}}_{\#4}}=5.05\;kNm^{-1}\;\pm \;0.93$. The model error assumes 3µm error on the lengths and 1% on *E*.

Similarly, for design #2, results are shown in Figure 14 using Equation (2). The stiffness is measured to be $4.86\;kNm^{-1}\;\pm \;0.16$ for sample # 4 and $4.33\;kNm^{-1}\;\pm \;0.23$ for sample # 6. All these values are in line with the theoretical predictions.

### 3.3. Optomechanical Results for Design #2

**Figure 15:**
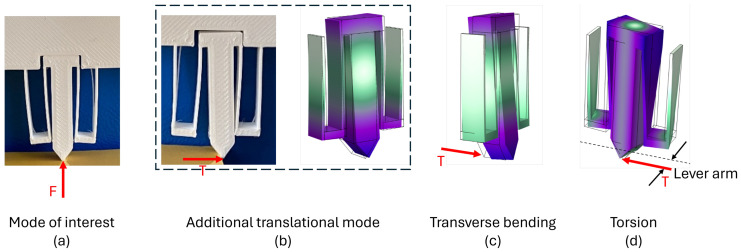
Beside the deformation mode of interest for the measure (**a**) and the additional translational (**b**) exhibiting a very close stiffness, two other modes could parasitize the measure in case of misalignment of the loading direction with respect to the sensor: transverse bending (**c**) and torsion (**d**).

While design #1 is necessarily subject to a transverse force *T* generated by the indented medium due to the lateral displacement of its indentation tip (which may modify the expression of the stiffness $k_{1}$), design #2 is free of transverse force solicitation for perfectly aligned force *F* (shown in Figure 15a). However, even with design #2, a small angular misalignment between the sensor axis and the loading direction may occur, and this may affect the response of such a compliant structure, deforming the sensor along an additional translational mode very close to the primary one in terms of stiffness (shown in Figure 15b). A countermeasure for the future could therefore foresee a waveguide (and its Bragg grating) in both the left and right arms of design #2, in such a way that the sum of left and right strain signals could lead to *F* and the difference in signals could lead to *T* (see complements in Appendix B). This approach is not possible with design #1, and, therefore, only design #2 has been considered in the following.

In addition to the limits of this work, transverse bending (Figure 15c) and torsional (Figure 15d) deformation should also be considered. These modes are clearly stiffer as estimated from numerical mode analysis (the transversal mode exhibits a resonance frequency 2.5 times higher than the targeted working mode, and the torsional mode 3.8 times larger). The torsional mode is unlikely to be activated since it requires a lever arm, which would vanish when manufacturing the tip as a sphere with (almost) punctual contact. Still, a more detailed study should be carried out, especially concerning the transverse bending mode.

For design #2, two different replicates of the sensor have been used (samples 4 and 6) in two different days. The first replicate was tested twice, while the second replicate was tested four times.

During a given test, the structure was gradually deformed by imposing an increasing tip displacement $\delta_{y}$, further converted into the corresponding strain ϵ located on the Bragg grating, close to the cantilevered side (point *O*) at a distance from the neutral axis designed to be *x*_Bragg,th_ =12.5µm (see Section 4 and the discussion on sources of errors for further explanations on *x*_Bragg,th_):

(8) $$\epsilon =\frac{x_{\mathrm{Bragg},\mathrm{th}}\delta_{y}}{sL}$$

where *s* and *L* are the values measured for samples 4 and 6 as indicated in Table 1. For each applied load, the optical spectrum was processed as indicated in Section 2.4.2, and the wavelength shift could be evaluated. The linear relationship between the shift (in pm) and the strain (expressed in microstrain, the standard scale in the field expressing 0.1% deformation as 1000 microstrain) is a property of Bragg reflection, and the slope is a material property, i.e., 1.24pm/µϵ for glass. Initially considering *x*_Bragg,th_ =12.5µm as designed, we found a slope of 0.82pm/µϵ. Assuming an effective value *x*_Bragg,eff_ different from *x*_Bragg,th_ =12.5µm, *x*_Bragg,eff_ has therefore been fitted to match the theoretical slope for the glass, equal to 1.24pm/µϵ.

**Figure 16:**
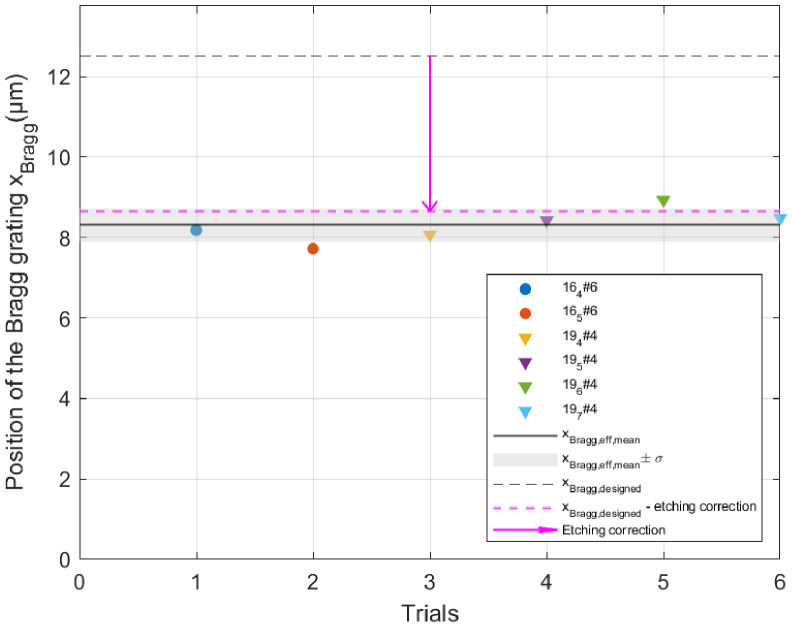
Each of the six trials lead to a different value of $x_{\mathrm{Bragg},\mathrm{eff}}$, accounting for the misalignment of the optical fiber and the complex interaction between the light beam and the Bragg grating in a non-uniform strain field. The chosen value at design is *x*_Bragg,th_ =12.5µm, the average *x*_Bragg,eff_ =8.3µm and the related standard deviation σ=0.4µm. The etching correction of 3.85µm is explained in the next section.

**Figure 17:**
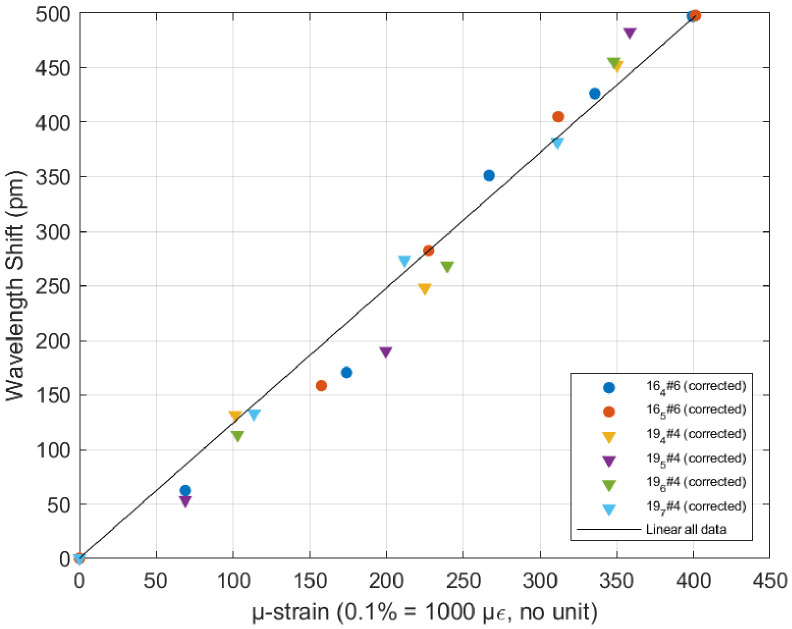
Wavelength shift in the reflected light as a function of the applied strain.

A different fit is led for each trial (see Figure 16 and details in Appendix E), leading to the value *x*_Bragg,eff_ =8.3µm ± 0.4, which was further used in Figure 17, since we have no other experimental mean to assess the true value of $x_{\mathrm{Bragg}}$.

Next, the displacement $\delta_{y}$ was converted into the corresponding applied force using the stiffness $k_{2}$:

(9) $$F=k_{2}\delta_{y}$$

**Figure 18:**
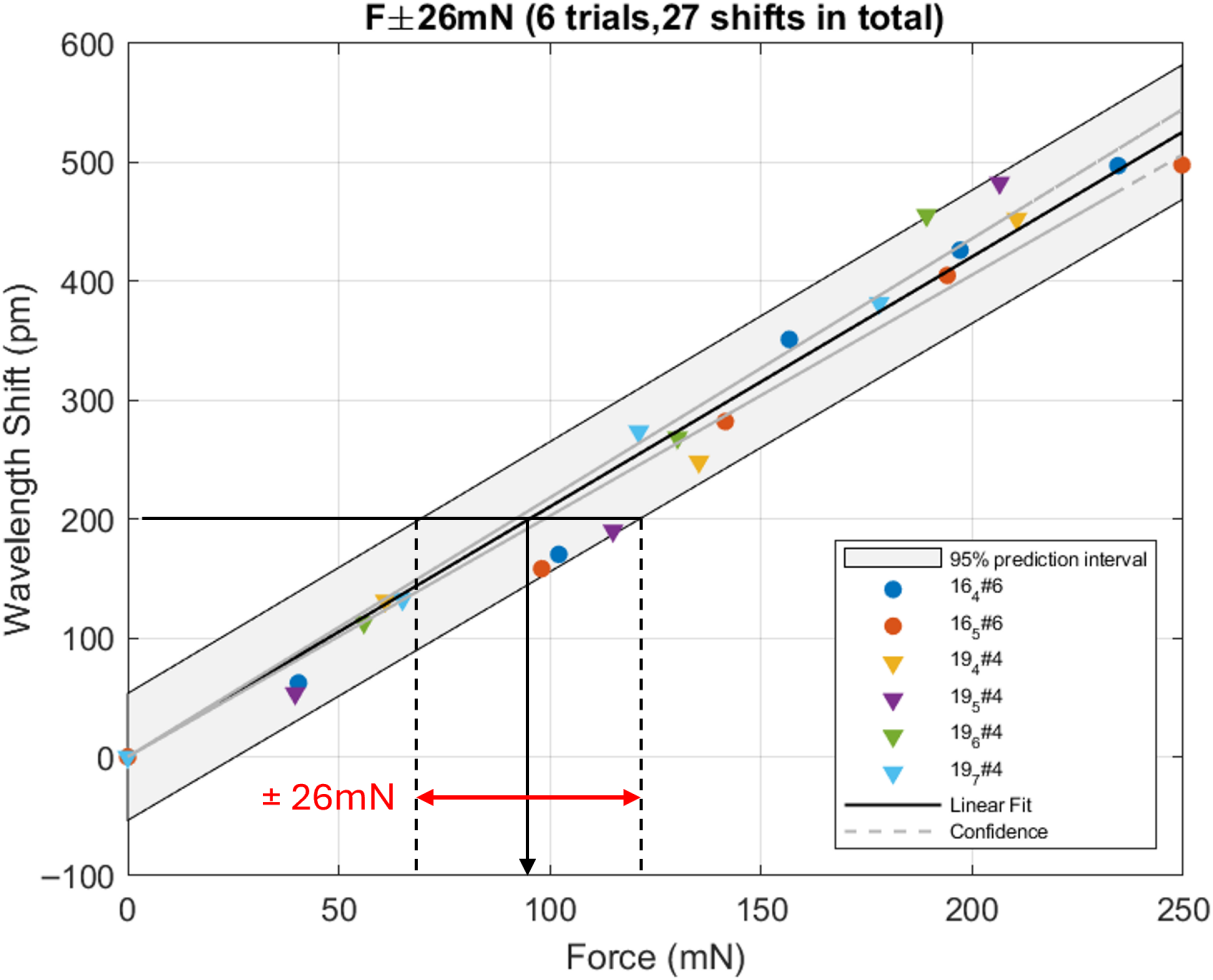
Wavelength shift of the reflected light as a function of the applied force. Due to the noise, any future shift could be converted into a force with an uncertainty interval ±26mN.

**Figure 19:**
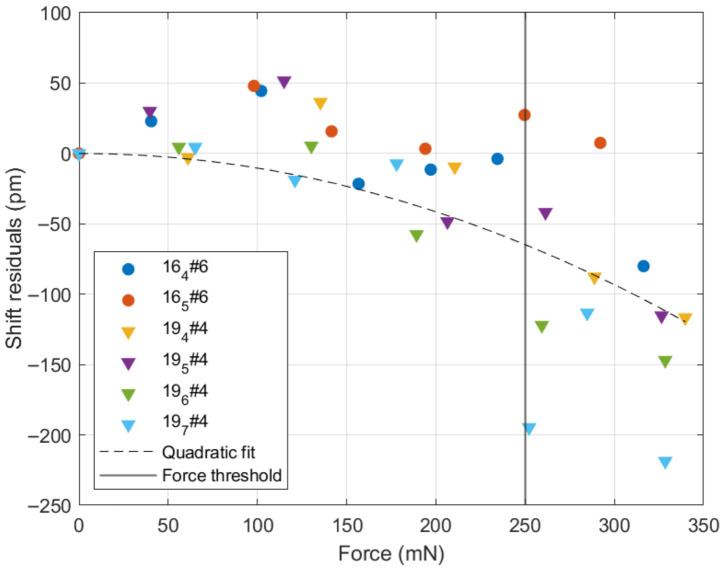
Shift residual as a function of the applied force. These residuals are not perfectly random since a quadratic trend as a function of the applied force can be observed, with an identifiable threshold around 250mN above which the noise seems to increase. This threshold was used to limit the force values in the analysis.

The wavelength shift could then be plotted against the force as shown in Figure 18. The six different series of measures fairly collapse along the linear regression line, and the corresponding residuals are shown in Figure 19. On this latter figure, it can be shown that these residuals are not distributed randomly as expected when all relevant information has been extracted from the fit. The residuals instead grow quadratically with the applied force. We therefore limited our analysis to applied forces below 250mN since this threshold seems to be a turning point in the evolution of these residuals.

**Figure 20:**
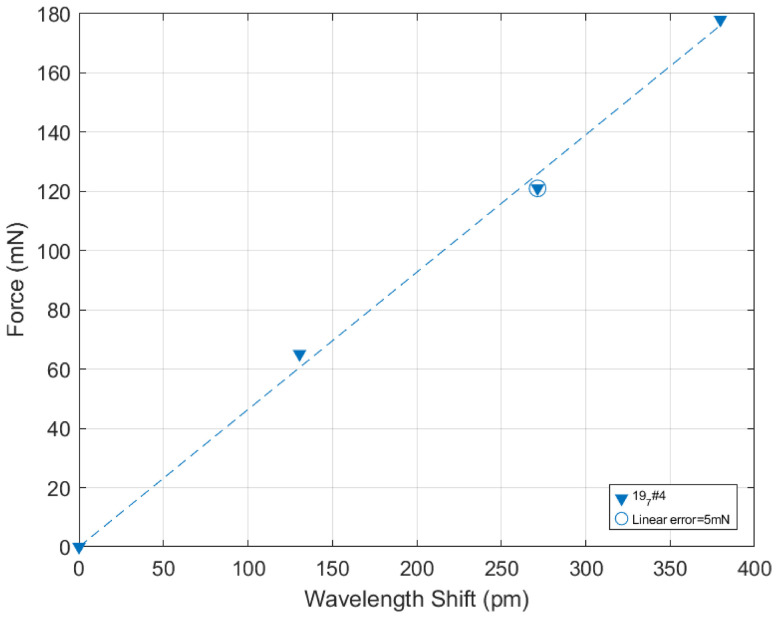
Force vs shift result for the $19_{7}\;\#4$ series of data: the force error is only 5mN, which is lower than 3% of the 180mN full range.

Let us, however, observe that up to now, the analysis has been based on the pooling of results obtained with two different sensors across six repetitions. If this provides a conservative and reliable estimation of the sensor’s performances, it can, however, be noted that in the best series of measures ($19_{7}\;\#4$), the force error is only 5mN, which is lower than 3% of the 180mN full range (Figure 20).

## 4. Discussion

**Figure 21:**
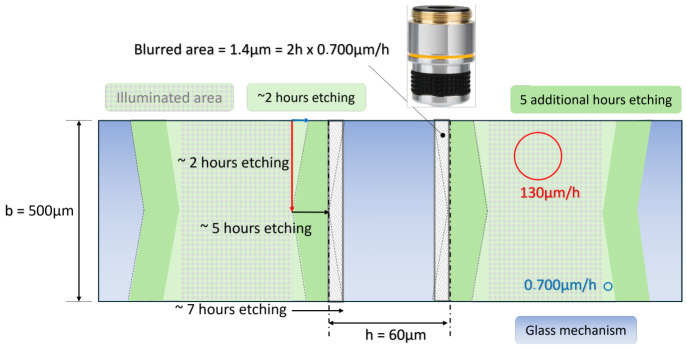
Sketch of the differential etching in the illuminated area vs the non-illuminated area. Vertical walls are therefore etched with a 1/200 slope, leading to a b=500µm thickness to a blurred zone of about 1.4µm in the measure of lengths with the microscope.

The first source of error identified in this paper is the geometrical error measured with the microscope. On top of a typical uncertainty of 1µm/pixel, the parasitic etching illustrated in Figure 21 induces an additional error. Illuminated area typically undergoes an etching rate of about 130µm/h while non-illuminated areas are etched at a slower pace (200× slower, 0.700 µm/h). As a consequence, the etched walls cannot be perfectly vertical (i.e., perpendicular to the glass slide) and rather exhibit a 1/200 slope (exact value is 0.0054). For a slide thickness b=500µm, this means a blurred area of about 1.4 µm. We therefore consider 2.5µm uncertainty in the different lengths measured with the microscope.

**Figure 22:**
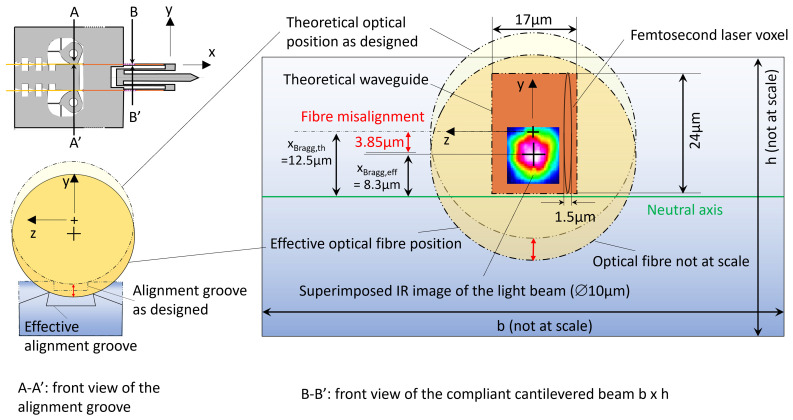
(**Left**) Front view A-A’ perpendicular to the propagation direction *x*: the differential etching rates lead to a fiber misalignment of about 3.85µm. (**Right**) representation of the waveguide geometry and the fiber misalignment in the front view BB’: the theoretical waveguide section is $17\times 24\;{µm}^{2}$, made of overlapping laser voxels $1.5\times 24\;{µm}^{2}$ separated with a 0.5µm pitch. The actual laser spot was imaged by [41] with an IR camera, unraveling an axially symmetric light beam of about 10µm. This IR image has been pasted onto this sketch to illustrate the misalignment between $x_{\mathrm{Bragg},\mathrm{th}}$ and $x_{\mathrm{Bragg},\mathrm{eff}}$ (adapted from [41]).

**Figure 23:**
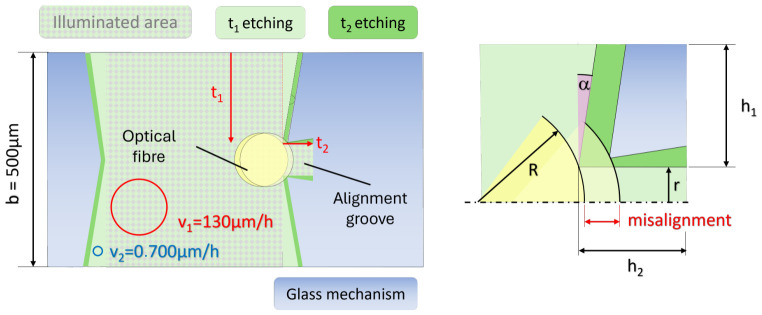
Due to differential etching rates in the illuminated vs. the non-illuminated area, the groove edges, which position the optical fiber with respect to the glass mechanism, can be misaligned (details are given in Appendix E).

A second source of error is related to the misalignment of the optical fiber with respect to the waveguide. Indeed, it should be noted that when the mechanism is loaded, the deflection $\delta_{y}$ is measured while the strain ϵ is derived. This calculation depends on the distance between the neutral axis and the actual location of the interaction between the light beam and the Bragg grating. As shown in Figure 22 (A-A’ front view), the alignment groove section exhibits a trapezoidal section instead of a rectangular one (see Figure 23 and details in Appendix F), with a widening of the slit and a bit of undercut. The B-B’ front view in Figure gives a representation of this misalignment with respect to the waveguide (orange rectangle) and the neutral axis (green line). This misalignment is estimated at 3.85µm (see details in Appendix F). Since this misalignment comes from systematic over-etching, as explained, compensation strategies could be implemented in the future, such as geometric pre-corrections based on our understanding of the etching process.

Let us mention that, besides the misalignment, the shape of the waveguide might also play a role. Since the Bragg grating is not made of infinitely small points and since the light beam is Gaussian (i.e., exhibits a spatial distribution of the power), the effective distance $x_{\mathrm{Bragg},\mathrm{eff}}$ between this interaction and the neutral axis can be different from the designed one $x_{\mathrm{Bragg},\mathrm{th}}$.

Two other sources of error should be accounted for, but have not been estimated in this work. The first one arises from Equation (6), since the error on $\delta_{y}$ is the sum of the errors on $\delta_{\mathrm{sensor}}$ and $\delta_{\mathrm{blade}}$, each of which is a few micrometers. Additionally, not only $\delta_{y}$ or ϵ are inaccurate: the shift estimation depends on the calculation of the Bragg wavelength, which might be impacted by birefringence as introduced in Figure 10. Such a birefringence effect is reported in the literature on fused silica specimens exposed to low-energy femtosecond pulses [42] and on fiber Bragg gratings [43,44]. This could arise from the anisotropy of the laser process: while denoting *x* the femto-second laser direction and *y* the light propagation axis, this birefringence could arise from anisotropy of optical properties along *x* and *z*. This assumption should be checked using polarized light in the future.

In this study, as well as in the targeted application, the number of cycles imposed on each sensor is supposed to be very low (<10 cycles). However, there is no stress threshold for which a glass mechanism would be 100% safe, similar to fatigue analysis with metals. The failure probability *P* is rather described with a so-called Weibull distribution. In reference [40] by Amez-Droz in 2024, for the same glass slides and the same manufacturing process, this failure probability P(σ) is given as a function of the mechanical stress σ by:

(10) $$P=1-e^{-{\left(\sigma /\sigma_{N}\right)}^{m}}$$

with $\sigma_{N}=1.95\;G\mathrm{Pa}$ and m=4.86. With a strain level at about $400\times 10^{-6}$, this work presents stress levels up to σ=28.8MPa, leading to a failure probability close to zero ($10^{-9}$).

Finally, the reasons unraveling the information remaining in the residuals of Figure 19 should be further investigated.

## 5. Conclusions

This paper reports the integrated fabrication of a monolithic force sensor made of a compliant glass mechanism, an interfacing technique to connect it to an optical fiber aligned with an internal waveguide, and a Bragg grating used to monitor the structure’s strain during mechanical loading. An extensive characterization has been achieved (geometry, stiffness, waveguide parameters, shift-force response, shift-strain response). The result is a force sensor with a range of about 250mN with a resolution of ±26 mN. A less conservative point of view on the same data can also be given while considering the $19_{7}\;\#4$ series, which exhibits a force error of 5mN, which is lower that 3% of the 180mN force range.

The perspectives are twofold. First, the resolution should be further increased by improving the reflected spectrum quality. Second, the shape of the indentation tip should be produced into classical geometries (cones, pyramids), allowing the application of post-processing methods relevant for viscoelastic soft biomaterials [45,46,47]. Obviously, the work reported in this paper is only a proof-of-concept on this long journey, and a much larger set of measures should be achieved to lift the statistical limits of the current dataset.

## Figures and Tables

**Table 1:** Geometrical properties as designed and measured for designs #1 and #2.

(All Sizes in µm)	b	h	L	s	Δx	Δy
Design #1 (as designed)	500	40	600	375	100	n.a.
Design #1 (sample)	498	35	606	378	106	n.a.
Design #2 (as designed)	500	60	1900	500	150	70
Design #2 (as measured, sample #4)	495	59	1905	501	144	68
Design #2 (as measured, sample #6)	499	59	1902	500	143	67

n.a. is not applicable.

**Table 2:** Summary of the process parameters.

	Energy (nJ)	Speed (mm/min)	Repetition Rate (kHz)	Pitch (µm)	Source
Fabrication	230	950	1000	3 (//voxel waist)	[40]
				7 (//voxel height)	[40]
Waveguide	150	50	1000	0.5	Appendix C
Bragg gratings	150	15	1000	0.5	[41]

**Figure A2:**
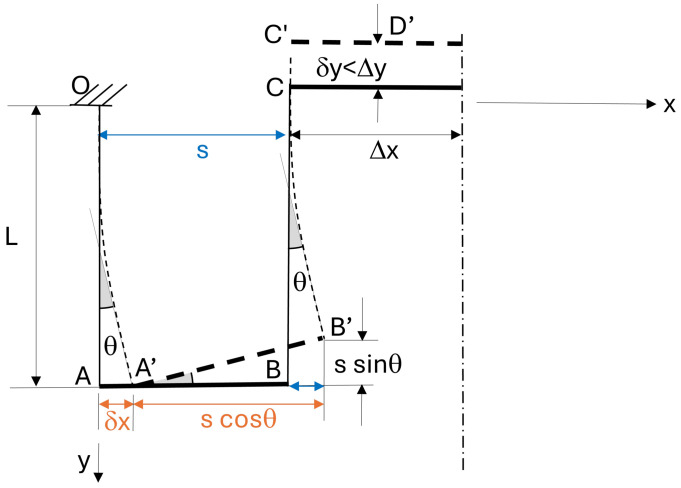
Kinematics model (design #2).

**Figure A4:**
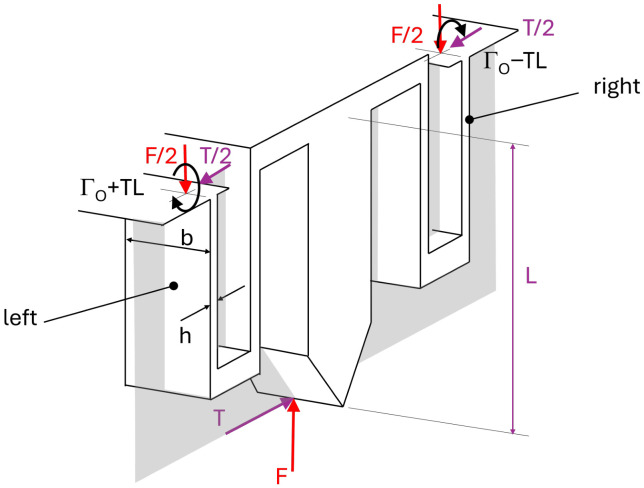
Sketch in presence of an additional transverse load *T* (in the plane of the mechanism).

**Figure A5:**
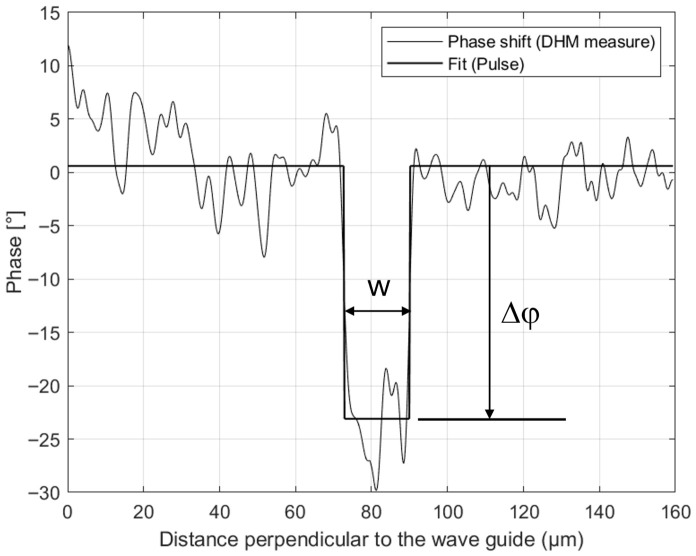
Evolution of the phase shift measured with digital holographic microscopy along a line crossing the waveguide (between 70µm and 90µm).

**Figure A6:**
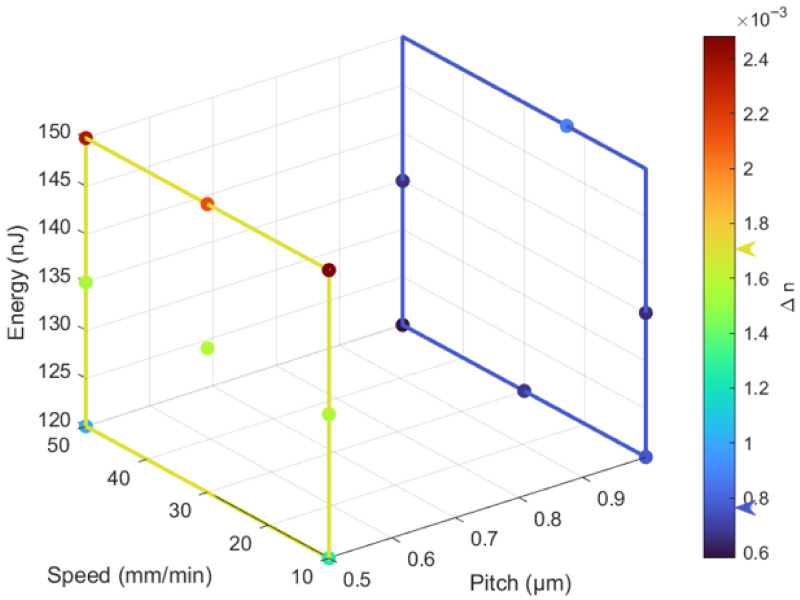
Scatter plot displaying the refractive index difference Δn for the different tested combinations of parameters.

**Figure A7:**
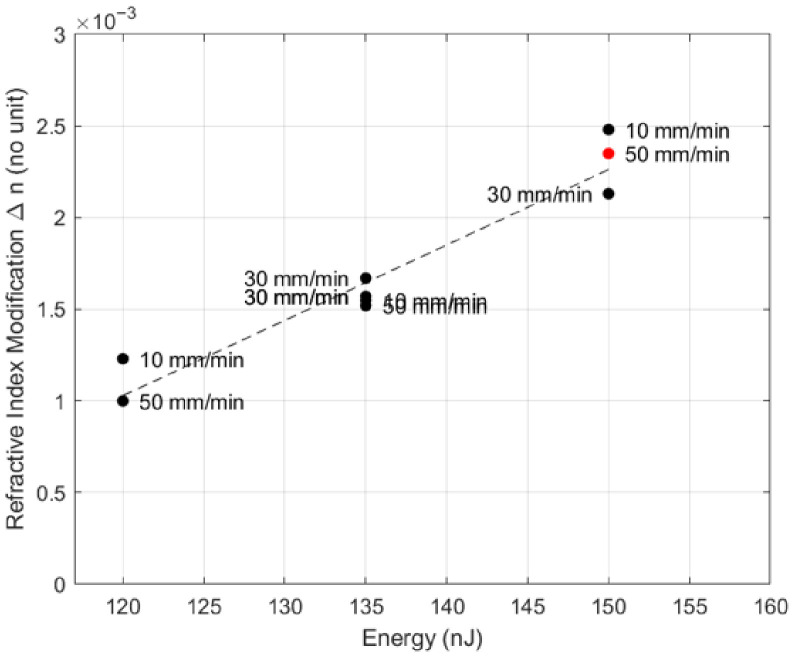
Since Δn is not significantly impacted by the writing speed, Δn can be plotted as a function of the pulse energy. For the value of 150nJ, the writing speed will then be selected at 50 mm/min to minimize the process duration.

**Figure A8:**
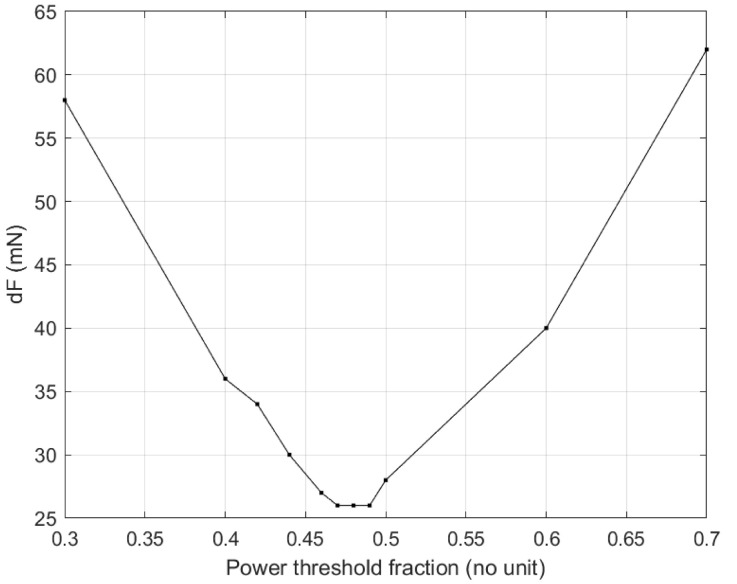
Evolution of dF the confidence interval on the force for η ranging from 0.3 to 0.7. The final value η=0.48 has been used to analyze the 27 experiments reported in this paper.

**Figure A9:**
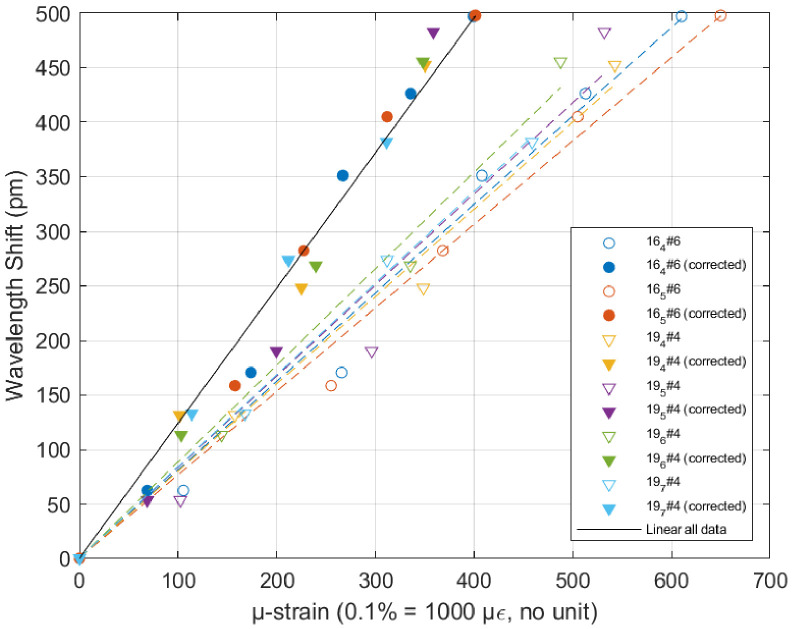
The mean slope of each of the six trials has been modified by adapting the value of $x_{\mathrm{Bragg},\mathrm{eff}}$ from the theoretical value $x_{\mathrm{Bragg},\mathrm{th}}=12.5\;µm$ to a value $x_{\mathrm{Bragg},\mathrm{eff}}$ leading to the slope 1.24 pm/µϵ.

**Figure A10:**
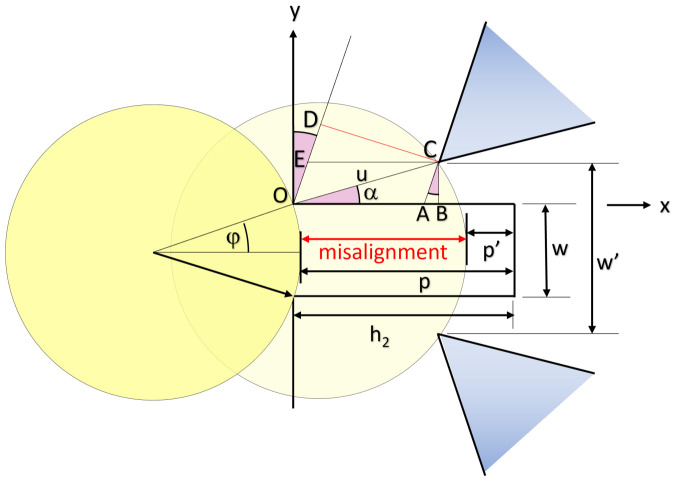
Error groove details. The darker yellow disk figures out the theoretical fiber position, pressed against a rectangular positioning slit. Due to differential etching, this slit is likely to be different, with a geometry sketched by the two blue prisms (triangles). This figure enables the geometrical calculations of the misalignment as a function of *w*.

**Figure A11:**
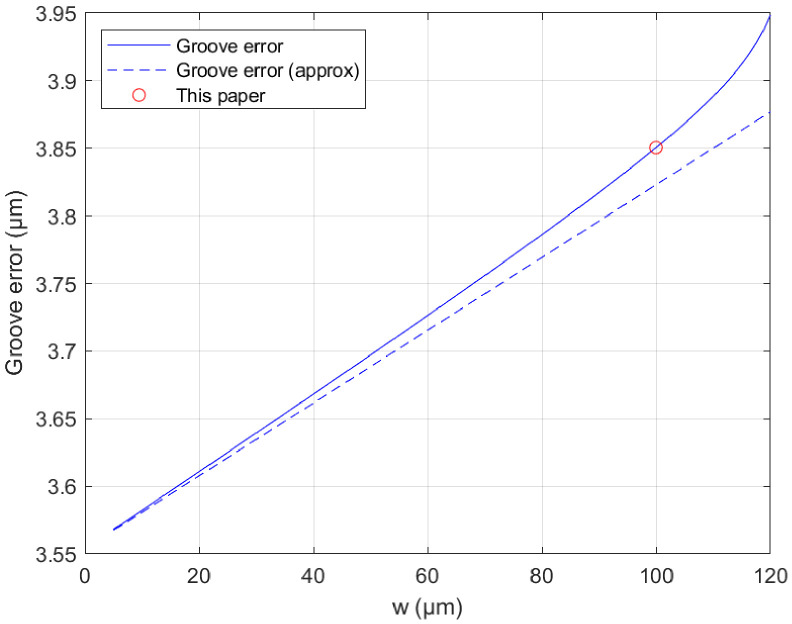
Due to the parasitic etching of non-illuminated area, the groove which is used to align the optical fiber undergoes induces a systematic alignment offset between 3.55 and 3.95 µm. In this paper, the groove width was 100µm, leading to 3.85µm misalignment.

## Data Availability

Data are available on request to the corresponding author.

## Appendix A. Mechanical Design (Design #1)

As sketched in Figure A1, the bending moment $M_{P}\left(y\right)$ in the section *P* located in x=0,y is constant and given by $M_{P}\left(y\right)=Fs$. Therefore, according to Euler–Bernoulli law for small displacements (i.e., $x^{\prime }<<$ which also means θ≈tanθ):

(A1) $$\begin{array}{ccc} x^{\prime \prime }\left(y\right) & = & \frac{Fs}{EI} \end{array}$$

(A2) $$\begin{array}{ccc} x^{\prime }\left(y\right) & = & \frac{Fs}{EI}y+\alpha \end{array}$$

(A3) $$\begin{array}{ccc} x(y) & = & \frac{Fs}{EI}\frac{y^{2}}{2}+\beta \end{array}$$

where α=β=0 considering the boundary conditions in *O*. The displacement $\delta_{x}$ and the slope θ in point *A* are therefore equal to:

(A4) $$\begin{array}{ccc} \delta_{x} & = & \frac{Fs}{EI}\frac{L^{2}}{2} \end{array}$$

(A5) $$\begin{array}{ccc} \theta (y=L) & = & \frac{Fs}{EI}L \end{array}$$

Finally, the displacement of point *C* can be found as a rotation of angle θ about the point *I* located at the intersection between the tangent to the deformed beam and the vertical axis *y*. The length $CC^{\prime }$ is equal to uθ where usinα=s. Hence:

(A6) $$\delta_{y}=u\theta \sin\alpha =s\theta =\frac{Fs^{2}L}{EI}$$

and the vertical stiffness $k_{1}$ is given by:

(A7) $$k_{1}=\frac{F}{\delta_{y}}=\frac{EI}{s^{2}L}$$

whose model error is given by:

(A8) $$\frac{dk_{1}}{k_{1}}=\frac{\Delta E}{E}+\frac{\Delta b}{b}+3\frac{\Delta h}{h}+2\frac{\Delta s}{s}+\frac{\Delta L}{L}$$

which can reach up to 20% with a typical error of 2µm error on lengths.

## Appendix B. Mechanical Design (Design #2)

### Appendix B.1. Kinematic Equations

Under the action of *F*, the mechanism (only a half is shown in Figure A2), OA is bent rightwards. Under the assumptions of linear assumptions (Hookean materials, small strain, small displacement), $AA^{\prime }$ can be considered to be perpendicular to OA: let us denote $∥\vec{AA^{\prime }}∥=\delta_{x}$. The bending angle of the cantilevered beam in *A* is noted θ. $\vec{AB}$ therefore makes the same angle θ with $\vec{A^{\prime }B^{\prime }}$. Expressing the horizontal distance between *A* and $B^{\prime }$ on two different ways:

(A9) $$\delta_{x}+s\cos\theta =s+\vec{BB^{\prime }}\;\cdot \;{\bar{1}}_{x}$$

and considering the linear approximation cosθ≈1, it can therefore be concluded that:

(A10) $$\vec{BB^{\prime }}\;\cdot \;{\bar{1}}_{x}=\delta_{x}$$

Similarly, we obtain the vertical displacement of *B* as:

(A11) $$\vec{BB^{\prime }}\;\cdot \;{\bar{1}}_{y}=s\sin\theta =s\theta \equiv \delta_{y}$$

### Appendix B.2. Equilibrium Equations

The successive three forces diagrams shown in Figure A3a–c are used to express the equilibrium conditions, leading to:

$$\begin{array}{ccc} \Gamma_{A} & = & \Gamma_{O}+O_{x}L\\ \Gamma_{B} & = & \Gamma_{O}-\frac{F}{2}s+O_{x}L\\ \Gamma_{C} & = & -\Gamma_{O}+\frac{F}{2}s \end{array}$$

and the bending moments $M_{OA}$ (resp. $M_{AB}$) in an arbitrary section of beam OA (resp. AB) can be expressed as:

$$\begin{array}{ccc} M_{OA} & = & \Gamma_{O}+O_{x}y\\ M_{BC} & = & -\Gamma_{O}-O_{x}y+\frac{F}{2}s \end{array}$$

### Appendix B.3. Euler–Bernoulli Equations

Applying the well-known Euler–Bernoulli equation 1/ρ=M/EI to beams OA and BC while making use of the linear assumption to express the curvature 1/ρ as:

(A12) $$1/\rho =\frac{x^{\prime \prime }}{{\left(1+x^{\prime 2}\right)}^{3/2}}\approx x^{\prime \prime }$$

we obtain successively:

(A13) $$\begin{array}{ccc} x_{OA}^{\prime \prime } & = & \frac{\Gamma_{O}}{EI}+\frac{O_{x}}{EI}y \end{array}$$

(A14) $$\begin{array}{ccc} x_{OA}^{\prime } & = & \frac{\Gamma_{O}}{EI}y+\frac{O_{x}}{2EI}y^{2} \end{array}$$

(A15) $$\begin{array}{ccc} x_{OA} & = & \frac{\Gamma_{O}}{2EI}y^{2}+\frac{O_{x}}{6EI}y^{3} \end{array}$$

where the integration constants are zero considering the boundary conditions in *O*: $x_{OA}\left(y=0\right)=0$ and $x_{OA}^{\prime }\left(y=0\right)=0$. Similarly:

$$\begin{array}{ccc} x_{BC}^{\prime \prime } & = & \frac{-\Gamma_{O}+Fs/2}{EI}-\frac{O_{x}}{EI}y\\ x_{BC}^{\prime } & = & \frac{-\Gamma_{O}+Fs/2}{EI}y-\frac{O_{x}}{2EI}y^{2}\\ x_{BC} & = & \frac{-\Gamma_{O}+Fs/2}{2EI}y^{2}-\frac{O_{x}}{6EI}y^{3} \end{array}$$

Expressing now $\delta_{x}=x_{OA}\left(y=L\right)=x_{BC}\left(y=L\right)$ and $\theta =x_{OA}^{\prime }\left(y=L\right)=x_{BC}^{\prime }\left(y=L\right)$, we obtain:

$$\begin{array}{ccc} O_{x} & = & 0\\ \Gamma_{O} & = & \frac{Fs}{4} \end{array}$$

### Appendix B.4. Stiffness

The stiffness $k_{2}$ is the ratio between the applied force *F* and the displacement $\delta_{y}=s\theta$:

(A16) $$k_{2}=\frac{F}{\delta_{y}}=\frac{4EI}{s^{2}L}$$

whose model error is given by:

(A17) $$\frac{dk_{2}}{k_{2}}=\frac{\Delta E}{E}+\frac{\Delta b}{b}+3\frac{\Delta h}{h}+2\frac{\Delta s}{s}+\frac{\Delta L}{L}$$

which can reach up to 12% with a typical error of 2µm error on lengths.

### Appendix B.5. Alternative Approach

Upon the application of the force *F*, the mechanism displaces $\delta_{y}$ and stores elastic energy given by:

(A18) $$W=\frac{1}{2}k_{2}\delta_{y}^{2}$$

This energy is stored in the bending of the 4 beams:

(A19) $$W=4\frac{1}{2}K_{M\theta }\theta^{2}$$

where $\delta_{y}=s\theta$ has been established previously and $K_{M\theta }=\frac{EI}{L}$ is given in [48]. Consequently:

(A20) $$k_{2}=4K_{M\theta }\frac{\theta^{2}}{\delta_{y}^{2}}=\frac{4EI}{s^{2}L}$$

### Appendix B.6. In Case of Transverse Force

If case of loading misalignment, a non symmetric load *T* (see Figure A4) could break the symmetry assumed here above, with two consequences:

- the clamping torque $\Gamma_{O}$ is augmented with TL/2 on the left and decreased with TL/2 on the right;
- the clamping force $O_{x}$ is not equal to zero anymore, but is equal to −T/2 on the left and +T/2 on the right.

As a consequence, using the expression of the displacement $x_{OA}$ in (A15) and expressing it for y=L gives the deflection on the left:

(A21) $$\delta_{x,\mathrm{left}}\left(y=L\right)=\left(\frac{Fs}{4}+\frac{TL}{2}\right)\frac{L^{2}}{2EI}-\frac{TL^{3}}{12EI}$$

and the deflection on the right:

(A22) $$\delta_{x,\mathrm{right}}\left(y=L\right)=\left(\frac{Fs}{4}-\frac{TL}{2}\right)\frac{L^{2}}{2EI}+\frac{TL^{3}}{12EI}$$

Since the strain in the left beam is proportional to $\delta_{x,\mathrm{left}}\left(y=L\right)$ and the strain in the right beam is proportional to $\delta_{x,\mathrm{right}}\left(y=L\right)$, *F* and *T* could be decoupled by summing and substracting signals from both the left and the right beams. This has not been implemented in this work and would require separated waveguides and Bragg gratings in both left and right beams.

## Appendix C. Waveguide Inscription Parameters

The waveguide quality is directly related to the contrast of refractive index inside and outside the guide. The machine parameters likely to have an impact on this contrast are the energy (nJ), the speed (mm/min) and the so-called pitch parameter (µm). For a triplet of these parameters, we can image the refractive index modification with a digital holographic microscope (Lyncee Tech, transmission) since such microscopy is based on the phase shift Δφ difference between a reference light beam (crossing the glass slide) and a second beam passing across the patterned waveguide (Figure A5).

The refractive index difference Δn can then be calculated as:

(A23) $$\Delta n=\frac{\lambda \varphi }{2\pi \Delta h}$$

where λ is the wavelength of the emitting laser (638 nm), Δh=24µm is the waveguide height estimated by [41] (see also Figure 22).

The impact of these printing parameters can now be evaluated while ranging as indicated in Table A1. The 18 combinations are shown in the scatter plot of Figure A6 where it can be immediately seen that Δn is much smaller for a pitch equal to 1µm (whatever the values of the speed and energy).

**Table A1:** Parameter’s range analyzed to find the optimal combination of parameters for waveguide impression with a femtosecond laser.

Parameter	Type of Variable	Low	High
Energy pulse	Continuous	120 nJ	150nJ
Writing speed	Continuous	10 mm $\min^{-1}$	$50\;\mathrm{mm}\;\min^{-1}$
Pitch	Discrete	0.5 µm	1µm

A deeper statistical analysis (with the software Statease/Design Expert) reveals that the influence of speed is not really significant. The speed will therefore be selected at its larger value to speed up the fabrication process. Lastly, Figure A7 shows the influence of the energy on Δn (for a pitch equal to 0.5µm), confirming the absence of significant effect of the speed. As a result, waveguides have been produced with an energy of 150 nJ, a speed of 50 mm/min and a pitch of 0.5µm.

## Appendix E. Position of the Bragg Grating

For each of the six trials, the experimental points located in Figure A9 have been linearly fitted:

(A24) $$\mathrm{shift}=a_{0}+a_{1}\mathrm{strain}$$

The resulting slope is an indication of the effective value of the distance $x_{\mathrm{Bragg},\mathrm{eff}}$ between the neutral fiber and the Bragg grating. Since this slope should be equal for glass to 1.24 pm/µϵ:

(A25) $$x_{\mathrm{Bragg},\mathrm{eff}}=x_{\mathrm{Bragg},\mathrm{designed}}\frac{a_{1}}{1.24\;pm/µ\epsilon }$$

Next, the most likely strain can be calculated as:

(A26) $$\epsilon \left(j\right)=x_{\mathrm{Bragg},\mathrm{eff}}\left(j\right)\frac{\delta y}{sL}$$

for each of the *j* trials. The 6 values of $x_{\mathrm{Bragg},\mathrm{eff}}$ are displayed in Figure 16.

## Appendix F. Groove Etching Error

Figure A10 is a zoom of Figure 23, which can be used to evaluate successively:

(A27) $$CD=v_{2}t_{2}=v_{2}\left(t_{\mathrm{etching}}-t_{1}\right)=v_{2}\left(t_{\mathrm{etching}}-h_{1}/v_{1}\right)$$

where $v_{1}=130\;µm/h$, $v_{2}=0.7\;µm/h$ and $t_{1}$ is the time required to etch a layer of thickness $h_{1}$ ($t_{1}<t_{\mathrm{etching}}$).

Considering the position of *C* in the Oxy axis:

(A28) $$\bar{OC}=u\cos\alpha {\bar{1}}_{x}+u\sin\alpha {\bar{1}}_{y}$$

where the angle $\alpha \approx \tan\alpha =\frac{v_{1}}{v_{2}}=0.7/130=0.0054$, we can finally express:

(A29) $$OA=EC=\frac{v_{2}h_{2}}{v_{1}\cos\alpha }$$

Next, using the similarity in triangles OBC and CBA:

(A30) $$\tan\alpha =\frac{AB}{BC}=\frac{u\cos\alpha -OA}{u\sin\alpha }$$

and using the expression for OA, *u* can finally be obtained:

(A31) $$u=v_{2}\left(t_{\mathrm{etching}}-h_{1}/v_{1}\right)\frac{1}{\cos2\alpha }$$

and the corresponding value of OB:

(A32) $$OB=v_{2}\left(t_{\mathrm{etching}}-h_{1}/v_{1}\right)\frac{\cos\alpha }{\cos2\alpha }$$

Let us now express the designed distance *p* between the optical fiber and the bottom of the groove (with sinφ=w/(2R)):

(A33) $$p=h_{2}-R\left(1-\cos\varphi \right)$$

Similarly, the true/effective position $p^{\prime }$ after etching can be expressed as:

(A34) $$p^{\prime }=\left(h_{2}-u\cos\alpha \right)-R\left(1-\cos\varphi^{\prime }\right)$$

with $\sin\varphi^{\prime }=\left(w+2u\sin\alpha \right)/\left(2R\right)$.

Finally, the misalignment writes:

(A35) $$\begin{array}{ccc} p-p^{\prime } & = & R\sqrt{1-\frac{w^{2}}{4R^{2}}}-R\sqrt{1-\frac{{\left(w+2u\sin\alpha \right)}^{2}}{4R^{2}}}+u\cos\alpha \\ & = & R\sqrt{1-\frac{w^{2}}{4R^{2}}}-R\sqrt{1-\frac{{\left(w+2u\sin\alpha \right)}^{2}}{4R^{2}}}+v_{2}\left(t_{\mathrm{etching}}-h_{1}/v_{1}\right)\frac{\cos\alpha }{\cos2\alpha }\\ & \approx & v_{2}\left(t_{\mathrm{etching}}-h_{1}/v_{1}\right) \end{array}$$

where $h_{1}=\left(b-w\right)/2$.
